# Autophagy-Associated Shrinkage of the Hepatopancreas in Fasting Male *Macrobrachium rosenbergii* Is Rescued by Neuropeptide F

**DOI:** 10.3389/fphys.2018.00613

**Published:** 2018-05-24

**Authors:** Sirorat Thongrod, Chaitip Wanichanon, Wilairat Kankuan, Tanapan Siangcham, Suratchanee Phadngam, Federica Morani, Ciro Isidoro, Prasert Sobhon

**Affiliations:** ^1^Department of Anatomy, Faculty of Science, Mahidol University, Bangkok, Thailand; ^2^Laboratory of Molecular Pathology, Department of Health Sciences, Università del Piemonte Orientale “A. Avogadro”, Novara, Italy; ^3^Faculty of Allied Health Sciences, Burapha University, Chonburi, Thailand

**Keywords:** neuropeptide F, autophagy, starvation, hepatopancreas, giant freshwater prawns

## Abstract

Invertebrate neuropeptide F-I (NPF-I), much alike its mammalian homolog neuropeptide Y, influences several physiological processes, including circadian rhythms, cortical excitability, stress response, and food intake behavior. Given the role of autophagy in the metabolic stress response, we investigated the effect of NPF-1 on autophagy during fasting and feeding conditions in the hepatopancreas and muscle tissues of the male giant freshwater prawn *Macrobrachium rosenbergii*. Starvation up-regulated the expression of the autophagy marker LC3 in both tissues. Yet, based on the relative levels of the autophagosome-associated LC3-II isoform and of its precursor LC3-I, the hepatopancreas was more responsive than the muscle to starvation-induced autophagy. Injection of NPF-I inhibited the autophagosome formation in the hepatopancreas of fasting prawns. Relative to the body weight, the muscle weight was not affected, while that of the hepatopancreas decreased upon starvation and NPF-1 treatment could largely prevent such weight loss. Thus, the hepatopancreas is the reserve organ for the nutrient homeostasis during starvation and NPF-I plays a crucial role in the balancing of energy expenditure and energy intake during starvation by modulating autophagy.

## Introduction

Neuropeptide F (NPF), a member of the FMRFamide-related peptide group, plays an important role in the regulation of foraging, feeding-related behavior, circadian rhythm, stress responses, aggression, and energy homeostasis (Nassel and Wegener, 2011), and is therefore considered homologue to mammalian NPY. Invertebrate NPF was identified first in the flatworm *Moniezia expansa* (Maule et al., 1991). In *Drosophila*, NPFs consist of two types, NPF and short NPF (sNPF), and both function in the regulation of food intake and increase of body size (Lee et al., 2004; Carlsson et al., 2013). Two crustacean isoforms of NPF, i.e., NPF-I and NPF-II, have been identified in the penaeid shrimps *Litopenaeus vannamei* and *Marsupenaeus japonicus* (Christie et al., 2011). The two isoforms were shown to be differentially expressed in the nervous, midgut and muscle tissues of both species. Interestingly, supplementing the diet with the NPF-I promoted the food intake and the growth of juvenile *L. vannamei* (Christie et al., 2011). Recently, by transcriptomic analysis we identified three isoforms of NPF (MrNPF-I, II, III) and four isoforms of sNPF (sMrNPF) in the central nervous system (CNS) and the ovary of the female giant freshwater prawn *Macrobrachium rosenbergii* (Suwansa-Ard et al., 2015). More recently, we demonstrated that MrNPF-I expressed in the ovaries of *M. rosenbergii* could stimulate ovarian maturation by shortening the ovarian cycle (Tinikul et al., 2017). While NPF is known to promote food intake and organ growth, it is unknown how NPF affects tissue homeostasis under fasting conditions. Here, we asked how NPF would modulate autophagy in two nutrient-storing organs, i.e., hepatopancreas and muscle, of prawns subjected to chronic fasting. Autophagy (referring to macroautophagy) is a physiological process for the gross degradation of cytoplasmic and membranous structures within lysosomes that preserves organ and systemic homeostasis at critical times during development and in response to metabolic stress through balancing the sources of energy and of metabolites (Kroemer et al., 2010; Russell et al., 2014; Kaur and Debnath, 2015). The basic process starts with the formation of a double-membrane protrusion from the endoplasmic reticulum (so-called the phagophore) that expands to engulf the cytoplasmic components and finally closes up to form a double-membrane vesicle called autophagosome (Feng et al., 2014; Galluzzi et al., 2017). Subsequently, the autophagosome fuses with endosomal-lysosomal organelles to form the autolysosome, wherein the lysosomal hydrolases digest the engulfed components (Feng et al., 2014; Galluzzi et al., 2017). Eventually, the digested products are transported back to the cytoplasm for reuse in biosynthesis or production of energy to meet the needs of the cell (Ravikumar et al., 2010; Zhang and Baehrecke, 2015). Two main signaling kinases control autophagy: mTOR (the mechanistic target of rapamycin), that senses the availability of nutrients, and AMPK, that senses the lack of energy (Alers et al., 2012).

Amino acids activate mTOR, which in turn switches off autophagy through the inhibitory phosphorylation of UNC51-like kinase 1 (ULK1), ortholog of the autophagy-related gene 1 (*Atg1*) (Kim et al., 2011; Roach, 2011; Alers et al., 2012; Wong et al., 2013). On the other hand, reduced synthesis of ATP leading to AMP accumulation triggers AMPK, which inactivates mTOR while directly activating ULK1 (Kim et al., 2011; Roach, 2011; Alers et al., 2012; Wong et al., 2013). Downstream to the ULK1 complex (formed by ULK1/Atg1, Atg13, FIP200 and Atg101) is the class III PI3K (PI3KC3/Vps34) complex that includes Beclin-1, Vps15, and Atg14 (Stjepanovic et al., 2017). This complex produces the phosphatidylinositol-3-phosphate (PI3P) needed for membrane recruitment at the phagophore assembly site (Wirth et al., 2013; Nascimbeni et al., 2017). The elongation of the phagophore and the biogenesis of the mature autophagosome require the activity of two ubiquitin-like conjugation systems, respectively, based on the ubiquitin-like proteins Atg12 and Atg8 (Klionsky and Schulman, 2014). Atg12 is first activated by Atg7 and then attached to Atg5 and Atg16 with the help of Atg10. Atg8 (the equivalent of mammalian LC3, Light Chain 3) is first activated by Atg4 and thereafter conjugated to the lipid phosphatidylethanolamine by Atg7 and Atg3. This latter step is fundamental for the sequestration of the autophagy cargo and the closure of the nascent autophagosome (Lippai and Low, 2014). Normally, LC3 resides in the cytoplasm as microtubule-associated protein light chain 3 (MAP-LC3), also known as LC3-I isoform, and is inserted into the inner and outer membranes of the nascent autophagosome after post-translational lipidation, which generates the LC3-II isoform (Fullgrabe et al., 2014; Elizabeth, 2015; Wani et al., 2015; Gozuacik et al., 2017). Notably, the conversion of LC3-I into LC3-II is used to monitor autophagosome formation, and the presence of LC3-positive vacuoles is considered a valid indicator of autophagy (Klionsky et al., 2016). Metabolic stress, particularly caloric restriction (CR), can trigger the AMPK signaling cascade that initiates autophagy (Kroemer et al., 2010; Ferraresi et al., 2017). In mammalian CNS, CR could activate autophagy in the hypothalamic neurons through NPY (Aveleira et al., 2015a,b; Ferreira-Marques et al., 2016).

In arthropods, autophagy has been studied during the development of *Drosophila melanogaster* (McPhee and Baehrecke, 2009; Mulakkal et al., 2014). The silkworm *Bombyx mori* expresses Atg genes at the metamorphosis stage (Malagoli et al., 2010). In addition, numerous Atg genes are involved in the regeneration process of the midgut epithelium in *Acheta domesticus* (Rost-Roszkowska et al., 2010). In crustaceans, autophagy plays a protective role in the midgut epithelium of *Eubranchipus grubii* to prevent cell death during apocrine secretion (Rost-Roszkowska et al., 2012). Moreover, autophagy has been observed in the intestine and hepatopancreas of the freshwater shrimp, *Neocaridina heteropoda*, during cell death processes (Rost-Roszkowska et al., 2012; Sonakowska et al., 2016). Recently, our group reported the presence of several autophagy gene mRNAs in the transcriptome and the expression of the corresponding proteins in various tissues of the giant freshwater prawn *M. rosenbergii* (Suwansa-Ard et al., 2016). More recently, we showed that starvation could induce oocyte maturation in association with up-regulation of autophagy markers in the ovary tissue of the female prawns (Kankuan et al., 2017). Here, we investigated the effect of NPF on the modulation of autophagy in the hepatopancreas and muscle of the male giant freshwater prawn *M. rosenbergii* under feeding and starving conditions.

## Materials and Methods

### Experimental Animals

Small male giant freshwater prawns *M. rosenbergii*, weighing 30 ± 5 g, were purchased from a local farm in Suphan Buri Province, Thailand. They were maintained in rectangular plastic tanks at Department of Anatomy, Faculty of Science, Mahidol University. The prawns were kept under a photoperiod of 12 h:12 h light–dark with continuous aeration and fed *ad libitum* with commercial pellets (Sunshine, Bangkok, Thailand) once a day for 1 week before treatment.

### Experimental Design

For *in vivo* studies with NPF injection, the prawns with intermoult phase (prawns undergoing molting events were discarded) were divided into four groups of 24 animals each: prawns in the first group were injected with 0.1 M phosphate buffered saline (PBS) and fed normally; prawns in the second group were injected with 0.1 M PBS and starved; prawns in the third group were injected with NPF at 2.5 × 10^-7^ mole/prawn and fed; and prawns in the last group were injected with NPF 2.5 × 10^-7^ mole/prawn and starved. Each treated prawn was injected at days 0 and 4. Then, they were placed in individual basket and reared in a rectangular tank. Eight prawns from each group were sacrificed at days 1, 4 and 8, and the hepatopancreas and muscle were collected and stored in liquid nitrogen or fixed with 4% paraformaldehyde. All animal protocols and experimental designs strictly followed the regulations set by Ethics Committee on the Use of Laboratory Animals, Faculty of Science, Mahidol University.

### Hepato-Somatic (HSI) and Musculo-Somatic (MSI) Indices

Each prawn was weighed before the sacrifice. The whole hepatopancreas (HP) and the body muscle (MS) taken from the first segment of the pleopod to the telson were weighed individually. Then, the hepato-somatic index (HSI) was calculated using the formula (HP fresh weight/body weight of each prawn × 100). The musculo-somatic index (MSI) was calculated using the formula (MS fresh weight/body weight of each prawn × 100). The average HSI and MSI was expressed as mean ± SD.

### Tissue Preparation for Histological Examinations

The fixed tissues were dehydrated in a series of ethyl alcohol (70, 80, 90, 95, and 100%) for 1 h each. Then, they were immersed in xylene for 2 h, infiltrated with paraffin and embedded in paraffin blocks. The embedded tissues were cut at 5 μm thickness using a rotary microtome (Leica RM2235, Germany), placed onto silane-coated slides and stained with hematoxylin and eosin.

### Hematoxylin and Eosin Staining

The sections were deparaffinized in xylene, rehydrated through a graded series of ethanol and immersed in distilled water. Then, they were stained with Mayer’s hematoxylin and eosin and mounted in a mounting medium. Finally, the sections were observed under a Nikon E600 light microscope and photographed by a Nikon DXM 1200E digital camera (Japan).

### Western Blot Analysis

Frozen HP tissues were homogenized in a lysis buffer and sonicated. Then, the homogenized tissues were centrifuged at 12,000 × *g* for 30 min at 4°C, and the supernatant were collected into fresh tubes. SDS–PAGE and Western blot analysis were performed to identify LC3 protein in the hepatopancreas and muscle extracts. Protein concentration was measured with the Bradford reagent (Sigma-Aldrich, United States) and quantitated by spectrophotometry (NanoDrop 2000, Thermo Fisher Scientific, Wilmington, DE, United States). Samples (30 μg) were fractionated by 15% SDS–PAGE and the proteins transferred onto a PVDF membranes (Millipore Corporation). The gels were stained with Coomassie Brilliant Blue (Sigma-Aldrich, United States) to visualize the remaining proteins. The membranes were blocked from non-specific bindings by incubating with 5% skim milk in PBS for 1 h at room temperature. Then, they were incubated with anti-LC3B (Sigma-Aldrich, United States) at a 1:500 dilution at 4°C overnight. Next, the membranes were incubated with anti-tubulin (code T5168, Sigma-Aldrich, United States) at a 1:1,000 dilution at 4°C overnight, and then incubated with anti-rabbit IgG coupled to HRP at a dilution of 1:10,000 for 2 h at room temperature. For negative control, the primary antibody was omitted and the membranes were incubated with anti-mouse IgG coupled to HRP at a dilution of 1:10,000 for 2 h at room temperature. Then, they were washed extensively with 0.2% Tween in PBS (0.2% PBST). Immunoreactive bands were revealed by using a chemiluminescence detection kit (PerkinElmer, Waltham, MA, United States) and detected with a VersaDOC Imaging System (Bio-Rad Laboratories, Inc., Hercules, CA, United States). Intensity of the each band was estimated by densitometry using Quantity one software (Bio-Rad Laboratories, Inc., Hercules, CA, United States) and ImageJ software (1.46r). The intensities of LC3-I and LC3-II bands were normalized versus alpha-tubulin, and expressed as means ± SD. Histograms of relative intensities were plotted by using Prism 5 (GraphPad software, Inc., La Jolla, CA, United States).

### Statistical Analyses

The HSI, MSI and the normalized densities of LC3-I and LC3-II bands in each group were presented as means ± SD. The HSI, MSI, LC3-I/TUB, LC3-II/TUB, and LC3-II/LC3-I ratio in different groups within the same period of sampling were compared and analysed by two-way Analysis of Variance (ANOVA) using the software GraphPad Prism 5, and *P* < 0.05 was considered to be statistically different.

## Results

### Eight Days Fasting Causes a Slight Loss of Weight and the Treatment With NPF Elicits No Effects on the Weight of the Whole Organism

**FIGURE 1:**
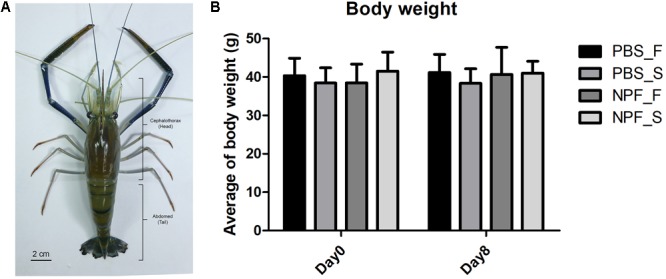
Effect of neuropeptide F on an anatomical structure and the body weight of *Macrobrachium rosenbergii*. **(A)** An example of small male freshwater prawns used in the experiment. **(B)** The average weights of the prawns in the control and NPF-injected groups at day 8 (no significant difference).

First, we took a general look at the systemic effects of prolonged starvation. In general, at day 8 the prawns subjected to starvation appeared less active. The body weight was slightly and not significantly affected by the lack of food (**Figure 1**): at day 8, the average weight of the prawns in the starvation group was about 10% lower than that of the control group (fed *ad libitum*). The treatment with NPF did not result in significant changes of the body weight, both in the fed and starved groups of prawns.

### Eight Days Fasting Causes Shrinkage of the Hepatopancreas Not of Muscle Tissue and Neuropeptide F Reverts the Effect

**FIGURE 2:**
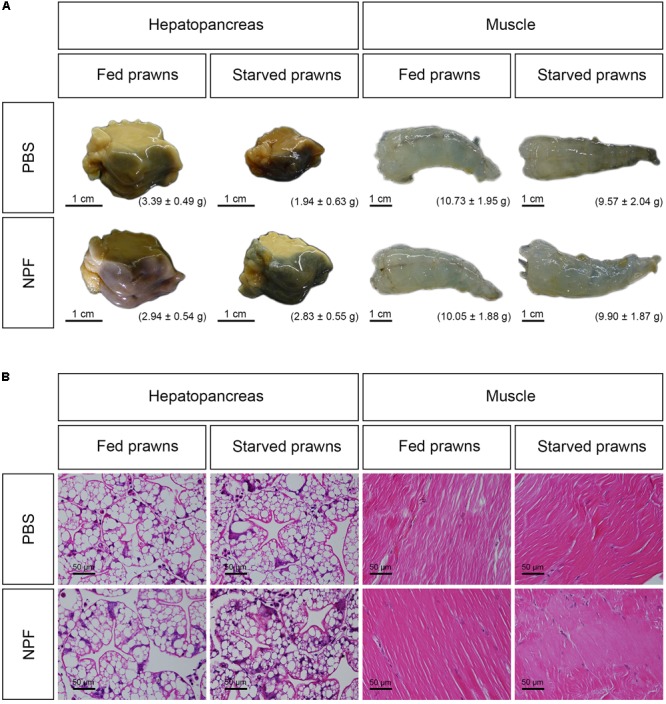
Effects of neuropeptide F and starvation on the structure of *M. rosenbergii* hepatopancreas and muscle at day 8. **(A)** Macroscopic pictures of the hepatopancreas and muscle **(B)** Photomicrographs of the hepatopancreas and muscle.

Next, we had a look at the effects of starvation and NPF treatments on specific organs. The macroscopic and microscopic morphologies of the hepatopancreas and of the muscle in control and treated groups at day 8 are shown in **Figure 2**. The size, weight and gross morphology of the muscle showed no changes among the groups. In addition, the muscle fibers of all treatment groups were not different from the control in terms of color and muscle banding at light microscopic level. In contrast, it is apparent that the size of the hepatopancreas decreased in the starved prawns: on average the weight was reduced by 42.77%. Worthy to note, the loss of weight and size in the hepatopancreas of starved prawns was largely prevented by NPF: on average, the weight of the hepatopancreas in starved prawns was reduced only by 3.74% when injected with NPF (**Figure 2A**). Moreover, the hepatopancreas in the starved prawns injected with NPF showed a higher number of R-cells, which are the nutrient reserve cells (**Figure 2B**).

**FIGURE 3:**
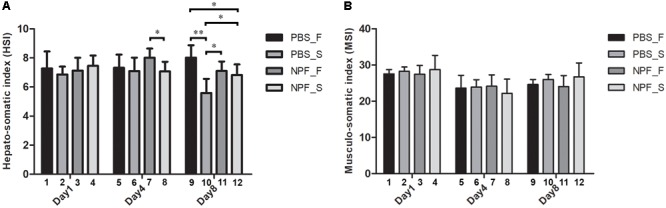
Effects of neuropeptide F and starvation on the hepatopancreas and muscle in male giant freshwater prawns. **(A)** Mean hepato-somatic index (HSI) of fed and starved prawns injected with PBS or NPF determined at days 1, 4, and 8. **(B)** Mean musculo-somatic index (MSI) of fed and starved prawns injected with PBS or NPF determined at days 1, 4, and 8. (^∗^*P* < 0.05, ^∗∗^P < 0.001).

To show these effects quantitatively, we calculated the HSI and the MSI, i.e., the percentage changes in the weight of the hepatopancreas and of the muscle versus the total body weight, respectively (**Figures 3A,B**). At day 1, the HSI showed no significant difference in all groups (**Figure 3A**, columns 1–4). At day 4, starvation caused no changes in the HSI (**Figure 3A**, columns 5–6), and the concomitant treatment with NPF only slightly reduced the HSI (**Figure 3A**, column 8 vs. 6). However, at this time point, the HSI was significantly decreased in the starved prawns injected with NPF compared to their counterparts fed *ad libitum* (*P* < 0.05) (**Figure 3A**, column 8 vs. 7). At day 8, starved prawns injected with PBS showed highly and significantly lower HSI than their fed counterparts (*P* < 0.001) (**Figure 3A**, column 10 vs. 9). Furthermore, HSI in starved prawns was lower than that in fed prawns injected with NPF (*P* < 0.05) (**Figure 3A**, column 10 vs. 11). NPF *per se* had no effects on HSI in fed prawns (**Figure 3A**, column11 vs. 9). NPF partially rescued the HSI in starved prawns (**Figure 3A**, column 12 vs. 10; *P* < 0.05), reaching values comparable to that of fed prawns (**Figure 3A**, column 12 vs. 9; *P* < 0.05). As expected based on gross morphology and size data of the muscle, no differences in the MSI values were observed between all groups of prawns at all time points (**Figure 3B**). These data suggest that the hepatopancreas, more than the muscle, suffers from the lack of nutrients and responds to NPF.

### Starvation Induces While NPF Inhibits Autophagy in the Hepatopancreas

To monitor autophagy, we followed the generation and accumulation of LC3, the hallmark of autophagosomes and autolysosomes (Klionsky et al., 2016). The cross-reactivity and specificity of the anti-human LC3B toward the prawn LC3 have been previously tested (Kankuan et al., 2017). The antibody recognizes both the cytosolic precursor (isoform LC3-I) and the vacuolar-associated mature isoform (LC3-II). A preliminary observation by immunofluorescence revealed an increased staining of LC3 in the hepatopancreas of starving prawns at day 8 compared to all other groups, while no significant changes of LC3 expression were observed in muscle tissue (data not shown).

**FIGURE 4:**
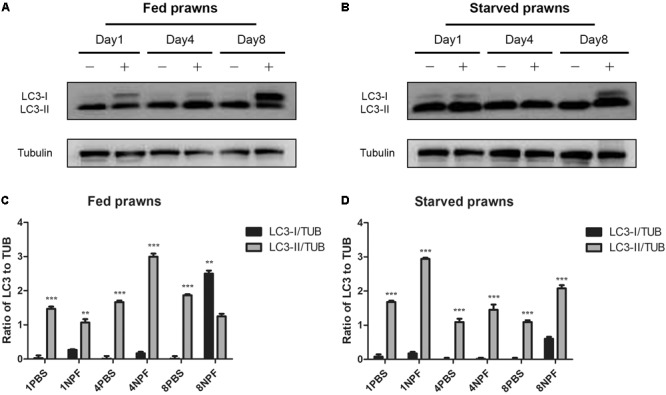
Effect of neuropeptide F on the expression of LC3 in the hepatopancreas. **(A)** Western blots of the autophagy markers, LC3I and LC3II, in the hepatopancreas of fed prawns injected with 0.1 M PBS (–) or neuropeptide F (+) at days 1, 4, and 8. **(B)** Western blots of LC3I and LC3II of starved prawns injected with 0.1 M PBS (–) or neuropeptide F (+) at days 1, 4, and 8. **(C)** Densitometric ratios of LC3 normalized with alpha tubulin of fed prawns from A (*N* = 8). **(D)** Densitometric ratios of LC3 normalized with alpha tubulin of starved prawns from B (*N* = 8) (^∗^*P* < 0.05, ^∗∗^*P* < 0.01, ^∗∗∗^P < 0.001).

To clearly assess the effects of starvation and of NPF treatments on the modulation of autophagy, we performed a western blotting of LC3 in the tissue homogenates. The cytosolic LC3-I isoform has an apparent molecular weight of 18 kDa, whereas the vacuolar-associated LC3-II isoform exhibits a higher electrophoretic mobility because it is conjugated with phosphatidylethanolamine, and shows an apparent molecular weight of 16 kDa (Klionsky et al., 2016). The relative intensities of LC3-I and LC3-II bands allow the quantitation of the autophagy process. In the hepatopancreas, LC3-II was expressed at higher level than LC3-I in all conditions (**Figure 4**), which indicates effective biogenesis of autophagic vacuoles. In fed prawns, NPF led to an accumulation of LC3-I in the hepatopancreas, especially at day 1 and even more evident at day 8 (**Figure 4A**; densitometry in panel C). This effect was mildly apparent at day 4. In the hepatopancreas of starving prawns (**Figure 4B**; densitometry in panel D), the expression of LC3-II was much higher compared to that in fed prawns, which is consistent with an overall up-regulation of basal autophagy. In these prawns, NPF still could elicit a partial block of autophagosome formation, as indicated by the accumulation of LC3-I, especially at days 1 and 8 (**Figure 4B**). Again, this effect was mild at day 4.

## Discussion

The autophagy machinery is ubiquitous in all living species, and it has been recently well characterized at genome level in crustaceans as well (Suwansa-Ard et al., 2016). We have recently described the autophagy response to starvation in the ovary of the female giant freshwater prawn *M. rosenbergii* (Kankuan et al., 2017). Interestingly, in this metabolic condition autophagy upregulation paralleled the development and maturation of the oocytes (Suwansa-Ard et al., 2016; Kankuan et al., 2017). Autophagy process is the mechanism for cell recovery in response to a variety of stresses, particularly in response to nutrient depletion (starvation) and caloric restriction (Kroemer et al., 2010; Ferraresi et al., 2017). In this study, we studied the modulation of autophagy in prawns subjected to starvation and to NPF, two metabolic stress conditions. In particular, we focused on the changes occurring in two specific organs, the hepatopancreas and the muscle. Induction of autophagy, as assessed by increased synthesis of LC3, was part of the general stress response to starvation in both organs. However, these effects were less prominent in the muscle (data not shown), consistent with lack of evident effects of starvation and NPF treatment on muscle homeostasis. In contrast, starvation and NPF greatly impacted on modulation of autophagy in the hepatopancreas, which was paralleled by changes in the organ size. The hepatopancreas, like vertebrate liver, helps maintain metabolic homeostasis through the steady supply of glucose and amino acids to the circulation or hemolymph in crustaceans. Therefore, during starvation the hepatopancreas is expected to compensate the lack of these nutrients by auto-digestion, which could be mediated by autophagy. Consistent with this interpretation, the HSI decreased along with time of starvation. Interestingly, administration of neuropeptide F largely lessened the shrinkage of HP in prawns subjected to starvation.

Neuropeptide F has been shown to stimulate feeding and growth in invertebrate species such as *Drosophila, Aplysia, Lymnaea*, and *Caenorhabditis elegans* (Wu et al., 2003; Jing et al., 2007; Nassel and Wegener, 2011). *M. rosenbergii* presents three isoforms of long NPF (MrNPF-I, NPF-II, and NPF-III) and four isoforms of short NPF (Suwansa-Ard et al., 2015). In the present study we used only the MrNPF-I. Previously, the effect on food intake of NPF I was investigated in several species. NPF-I has been reported to stimulate food intake and weight gain, whereas its knockdown reduced the food intake in both male and female *Schistocerca gregaria*, a desert locust (Van Wielendaele et al., 2013a). Interestingly, in adult female locusts, NPF injection also increased the size of oocytes (Van Wielendaele et al., 2013b). Furthermore, adult male locusts exhibited courtship behavior and increased gonadosomatic index (GSI) following NPF administration (Van Wielendaele et al., 2013c). In crustaceans, feeding with NPF-I-laced pellets increased the growth rate in juvenile *L. vannamei* (Christie et al., 2011). In addition, the female giant freshwater prawns, *M. rosenbergii*, injected with various concentrations of NPF showed increasing GSI and shortening of the ovarian maturation cycle (Tinikul et al., 2017). Strikingly, the effects of NPF on the stimulation of ovarian maturation resemble those of starvation that we reported in *M. rosenbergii* (Kankuan et al., 2017). It is tempting to hypothesize that NPF elicits these effects through induction of autophagy, much alike starvation.

Autophagy controls the liver metabolism to maintain metabolic homeostasis and is required also for muscle glucose homeostasis during physical exercise (He et al., 2012; Madrigal-Matute and Cuervo, 2016). Remarkably, the time-dependent modulation of starvation- and NPF-induced autophagy in the prawns differs in the hepatopancreas (which functionally corresponds to the liver in mammals) and in muscle. In our model, the muscle seemed to be less affected by starvation, based on the MSI. However, the fasting period used in our experiment might not be long enough to visibly exhaust the nutrient reserve capacity of the muscle to cause any appreciable decrease of MSI. Consistently, the level of LC3-II, which reflects the presence of autophagosomes and autolysosomes, was lower in the muscle than in the hepatopancreas tissue (data not shown). Thus, the hepatopancreas and muscle coordinately regulate the respective level of autophagy to face acute and chronic metabolic stresses.

In the hepatopancreas, LC3-II was generally higher than LC3-I, and NPF injection tended to increase the accumulation of LC3-I, especially at day 8. This implied that NPF enhanced the synthesis of LC3 in response to the metabolic stress, while limiting the conversion of LC3-I into LC3-II. Interestingly, the effect of starvation on *S. gregaria* showed higher levels of NPF transcription in starved animals compared to fed animals (Van Wielendaele et al., 2013a). In *Ruditapes philippinarum*, the mRNA expression of rp-NPF level was increased in the visceral ganglion after starvation and showed highest expression at 72 h. After refeeding, the rp-NPF level was declined immediately at 2 h (Wang et al., 2017). Furthermore, in rat cortical neurons, food depletion induced the increase of neuropeptide Y (NPY), a homolog of invertebrate NPF, in hypothalamic neurons which in turn caused autophagy expression (Aveleira et al., 2015b; Ferreira-Marques et al., 2016). Thus, it seems that a reciprocal regulation between the levels of autophagy and of NPF is fundamental to preserve organ homeostasis under fasting stress. In conclusion, the present study provided evidence that hepatopancreas and muscle of male giant freshwater prawns, *M. rosenbergii*, are the reserve organs that can support nutrient homeostasis during starvation through up-regulation of autophagy. Prolonged starvation caused important autophagy-associated shrinkage of the hepatopancreas, and NPF could prevent largely this effect by limiting autophagy.

## Ethics Statement

This study was carried out in accordance with ethical standards.

## Author Contributions

ST: data acquisition, data analysis and interpretation, and manuscript preparation. CW: data acquisition, manuscript preparation and final approval. WK: data analysis and interpretation. TS: data acquisition. SP: data interpretation. FM: data analysis. CI and PS: conception and design of study, writing and editing the manuscript and provided final approval.

## Conflict of Interest Statement

The authors declare that the research was conducted in the absence of any commercial or financial relationships that could be construed as a potential conflict of interest.
